# Shared Physiological Correlates of Multisensory and Expectation-Based Facilitation

**DOI:** 10.1523/ENEURO.0435-19.2019

**Published:** 2020-03-02

**Authors:** Stephanie J. Kayser, Christoph Kayser

**Affiliations:** 1 Department for Cognitive Neuroscience, Faculty of Biology, Bielefeld University, Bielefeld 33615, Germany; 2 Cognitive Interaction Technology, Bielefeld University, Bielefeld 33615, Germany

**Keywords:** cueing, EEG, expectation, multisensory, visual perception

## Abstract

Perceptual performance in a visual task can be enhanced by simultaneous multisensory information, but can also be enhanced by a symbolic or amodal cue inducing a specific expectation. That similar benefits can arise from multisensory information and within-modality expectation raises the question of whether the underlying neurophysiological processes are the same or distinct. We investigated this by comparing the influence of the following three types of auxiliary probabilistic cues on visual motion discrimination in humans: (1) acoustic motion, (2) a premotion visual symbolic cue, and (3) a postmotion symbolic cue. Using multivariate analysis of the EEG data, we show that both the multisensory and preceding visual symbolic cue enhance the encoding of visual motion direction as reflected by cerebral activity arising from occipital regions ∼200–400 ms post-stimulus onset. This suggests a common or overlapping physiological correlate of cross-modal and intramodal auxiliary information, pointing to a neural mechanism susceptive to both multisensory and more abstract probabilistic cues. We also asked how prestimulus activity shapes the cue–stimulus combination and found a differential influence on the cross-modal and intramodal combination: while alpha power modulated the relative weight of visual motion and the acoustic cue, it did not modulate the behavioral influence of a visual symbolic cue, pointing to differences in how prestimulus activity shapes the combination of multisensory and abstract cues with task-relevant information.

## Significance Statement

Perception can be enhanced by the combination of multisensory information and by the exploitation of amodal or symbolic cues presented within the same modality as the task-relevant information. We here asked whether the physiological correlates reflecting the behavioral benefits induced by each type of cue are similar or not. Using multivariate analysis of EEG data, we show that the perceptual enhancement induced by an acoustic cue and a visual-symbolic cue for the discrimination of visual motion arise from the same physiological source. This suggests that the impact of multisensory information and more abstract sensory expectations on perception arise from shared mechanisms.

## Introduction

Perception often benefits from additional information in addition to that made available by the primary and task-relevant features. One example is multisensory integration: often, our performance in detecting or discriminating stimuli is enhanced when the same information is presented in more than one sensory modality (Stein and Meredith, 1993; Kayser and Logothetis, 2007; Stein and Stanford, 2008). Here, perceptual benefits can arise both from the combination of partially redundant information, as in Bayesian fusion (Ernst and Bülthoff, 2004; Angelaki et al., 2009), or the auxiliary influence of one apparently irrelevant stimulus onto task performance in another modality (Jaekl and Harris, 2009; Caclin et al., 2011; Kim et al., 2012; Gleiss and Kayser, 2014b). As suggested by recent work, the neural mechanisms underlying multisensory benefits comprise a cascade of neural processes that commence with early multisensory influences in low-level sensory regions, continue with the automatic merging of multisensory information in parietal cortex, and culminate in the task- and context-dependent arbitration of different perceptual strategies of exploiting multisensory information in the frontal lobe (Rohe and Noppeney, 2015; Aller and Noppeney, 2019; Cao et al., 2019; Rohe et al., 2019).

From a behavioral perspective, similar benefits for perception also emerge from other types of auxiliary information, such as prior knowledge or expectations elicited by cues presented either within the same modality or in an amodal fashion (Summerfield and Egner, 2009; de Lange et al., 2013; Summerfield and de Lange, 2014). For example, priming participants to expect a specific sensory attribute boosts perceptual performance and reduces reaction times in discrimination tasks. One proposed mechanism by which expectation can improve perception is by an enhancement of the encoding of sensory information in low-level sensory cortices, in addition to post-sensory decision-level effects (Kok et al., 2012; Jiang et al., 2013; Cheadle et al., 2015; Bang and Rahnev, 2017; de Lange et al., 2018; Rungratsameetaweemana et al., 2018). The parallels between traditional cueing paradigms and studies investigating multisensory paradigms, in particular those focusing on auxiliary multisensory influences, raise the question of how far the neural mechanisms mediating the behavioral benefits arising from multisensory combination are the same as those mediating within-modality priming effects.

We here compare the physiological correlates of multisensory combination and within-modality cueing using EEG while human participants performed a visual motion discrimination task. Specifically, we capitalize on previous work that has established the cerebral correlates of multisensory combination in a task where acoustic motion enhances the discrimination of horizontal visual motion (Kayser et al., 2017; Kayser and Kayser, 2018): the physiological correlates of the perceptual benefit arising from congruent audiovisual stimuli in this task were shown to comprise the enhancement of visual motion encoding in (low-level) visual cortices at latencies of ∼300 ms post-stimulus onset. We here exploit this validated paradigm to ask whether the perceptual enhancement induced by a probabilistic within-modality visual cue arises from the same physiological processes that mediate the multisensory benefit.

Furthermore, we investigated the role of prestimulus activity in shaping the influence of task-relevant visual motion and the visual cue on behavior. Previous work has shown that multisensory perception is shaped by the state of oscillatory brain activity prior to a stimulus (Keil et al., 2012; Lange et al., 2013; Gleiss and Kayser, 2014b; Kaiser et al., 2019). For example, the overall tendency to combine visual and acoustic cues during temporal rate judgments was influenced by the level of prestimulus alpha power (Rohe et al., 2019). Given this link of prestimulus activity and multisensory combination, we asked whether between-modality multisensory integration and within-modality combination of a cue and stimulus are shaped in a similar manner by prestimulus activity.

## Materials and Methods

We report data obtained from a planned sample of 20 healthy adult participants (9 males; mean age, 23.4 ± 3.3 years). These adults participated after giving written informed consent and receiving a briefing about the purpose of the study. All had self-reported normal hearing and tested normal vision, declared no history of neurologic disorders, and were right handed (Oldfield, 1971). The study was conducted in accordance with the Declaration of Helsinki and was approved by the ethics committee of Bielefeld University. The sample size was decided a priori based on general recommendations for behavioral studies (Simmons et al., 2011). Data collection proceeded until the sample of 20 acceptable participants was obtained, whereby some (*n* = 7) participants dropped out because they either did not pass the sight test (one) or, during an initial screening session, exhibited a perceptual threshold for visual motion discrimination that was higher than an a priori set criterion of 25% motion coherence (six participants; see below).

### Experimental design and stimuli

**Figure 1.**
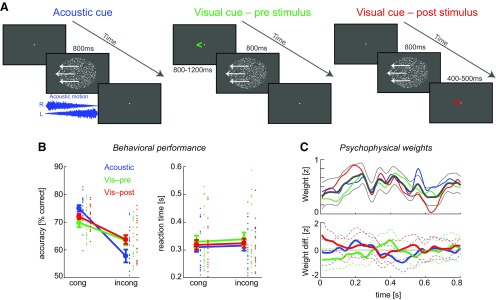
Paradigm and behavioral data. ***A***, Participants discriminated the direction of visual random-dot motion (800 ms stimulus duration). Across three conditions, the visual motion was accompanied by different auxiliary cues: simultaneous acoustic motion, a visual symbolic cue preceding the visual motion, or a visual symbolic cue following the motion stimulus. Each cue was congruent with the random-dot motion on 66% of trials. ***B***, For each cue type, response accuracy was higher for congruent trials, while reaction times (relative to a response cue) were not affected by congruency (mean and SEM across participants; dots represent single-participant data). ***C***, Psychophysical response templates (weights) were calculated to quantify the influence of the moment-by-moment motion energy on behavior. These templates were significant for most time points (top; thick black line, grand average; thin black line, 5% bootstrap confidence interval across conditions; thick colored lines, condition-wise group means), but were not affected by cue–stimulus congruence (bottom; think lines, condition wise group means; dashed lines, 5% bootstrap confidence intervals).

The task required participants to discriminate the direction (leftward or rightward) of random dot visual motion (Fig. 1*A*). The motion stimulus was presented following the onset of a fixation dot (800–1200 ms uniform delay), lasted 800 ms, and was presented on a 24 inch high-performance LCD monitor (catalog #PG279Q, ASUS) at 1920 × 1080 pixel resolution and a refresh rate of 120 Hz. The random dot pattern consisted of 1300 white, limited-lifetime dots that were presented on a neutral gray screen (16 cd/m^2^ background luminance) and covered 10° of visual angle (with the center 0.8° devoid of dots). Individual dots were 0.1° in diameter and moved at 4°/s. Each dot had a lifetime of 9 frames (at 120 Hz). A small percentage of dots moved coherently in the same direction (left or right). In an initial screening session, we determined the participant’s psychometric thresholds (∼71% correct performance) for discriminating motion directions using three interleaved 2-down, 1-up staircases. In the actual experiment, the motion coherence was additionally manipulated over the 800 ms (96 frames) of stimulus presentation: every period of 12 consecutive nonoverlapping frames was characterized by a different and independent coherence level (drawn from a normal distribution centered on the participant’s specific threshold with an SD of 10%, limited to the range 0–100%). This was performed to allow the quantification of perceptual weights, that is, how much each epoch of 12 frames contributed to the participant’s responses (see below). Across participants, the coherence thresholds were 18.2 ± 1.9% (mean ± SEM). From 400–500 ms following stimulus offset, a response cue was presented prompting the participants to respond. Given that some of the analyses reported here essentially reproduce a previous study (Kayser et al., 2017), it is important to note that the stimulus used here differed from that used in the previous study in that the motion coherence here changed randomly over the course of a single trial, while it was constant in that previous study. Also, the previous study used a speeded reaction time task, while we here used a fixed stimulus duration and a task emphasizing accuracy (see below).

The experiment comprised three conditions (Fig. 1*A*). In the first condition, visual motion was accompanied by a dynamic acoustic stimulus mimicking motion in either the same direction (“congruent”; 66% of trials) or the opposite direction (“incongruent”; 33% of trials) as the visual motion. Sounds were composed from white noise (at 44.1 kHz sampling rate) with an amplitude that was linearly modulated from 0 to the maximal level in opposite directions on left and right speakers over the stimulus period, inducing a percept of continuous acoustic motion (Meyer and Wuerger, 2001; Moore, 2003). Sounds were presented with a peak (P) amplitude of 65 dB(A) SPL rms level using speakers placed to the left and right of the monitor; onsets and offsets were cosine ramped (8 ms). In the second condition, visual motion was preceded by a symbolic visual cue that indicated the likely (66% correct) direction of the subsequent visual motion stimulus. The cue was presented during the entire fixation period. In the third condition, the visual motion stimulus was followed by a symbolic visual cue that indicated the likely (66% correct) direction of the preceding motion stimulus. The cue was presented starting 400–500 ms (uniform) following stimulus offset for a period of 500–600 ms. Participants completed 936 trials in total, 156 per motion direction and cue condition. Individual trials were separated by intertrial intervals of 1200–1500 ms (uniform) and were grouped into four experimental blocks, with each block comprising all three conditions. Within each block, groups of 78 subsequent trials featured the same condition (“mini-blocks”), with the three conditions (mini-blocks of 78 trials) appearing in pseudorandom order within each experimental block. Participants were instructed “to discriminate the direction of visual motion and to respond as quickly and accurately as possible following the response cue, while making use of the auxiliary information” by pressing a left or right arrow key on a keyboard (Das Keyboard) using the same hand for both keys.

During the experiment, monocular (right) eye movements were monitored using an EyeLink 1000 System (SR Research) at 250 Hz. For six participants, the system did not reliably track the eye, and the eye-tracking data could not be analyzed.

### Analysis of behavioral and eye-tracking data

From the behavioral responses, we calculated the percentage of correct responses and reaction times aligned to the response cue. For an analysis based on signal detection theory, we computed hit and false-alarm rates relative to leftward motion (Green and Sweets, 1966). To compute the response bias, we analyzed trials with the cue pointing to the left and right separately, and combined the bias measures (c), after converting both sides to have the same sign (Bang and Rahnev, 2017). We quantified the perceptual use of the moment-by-moment visual motion evidence using reverse correlation (Marmarelis, 1978; Eckstein and Ahumada, 2002). Specifically, we computed perceptual response templates (weights) relative to the two response options and normalized these within participants to *z* scores using a shuffling procedure (Neri and Heeger, 2002; Chauvin et al., 2005). These templates were computed based on the amount of motion evidence available in the random dot motion at each moment in time. This level of motion evidence was extracted *post hoc* from the single-trial motion stimulus based on algorithms previously used to detect horizontal motion using parameters suitable for the human visual system (Kiani et al., 2008; Urai et al., 2017). The resulting perceptual weights indicate how strongly (relative to chance) the visual motion influenced the participant’s response. We also modeled the participants’ single-trial choice using logistic regression, entering the visual motion stimulus, the cue, and their interaction with prestimulus power as predictors. Similarly, we modeled performance (response accuracy) based on prestimulus power and its interaction with motion–cue congruency.

From the eye-tracking data we extracted fixation events detected by the eye-tracking system (using the EyeLink 1000 “cognitive” setting) and computed the SD of all fixated positions along horizontal and vertical dimensions during stimulus presentation. In addition, we extracted saccadic eye movements, as detected by the EyeLink 100 system (velocity threshold, 30°/s; acceleration threshold, 8000°/s). We then counted the number of saccades exceeding an amplitude of 0.8° executed during the stimulus interval, hence excluding smaller microsaccades (Rolfs, 2009).

### Setup and EEG recordings

The experiment took place in a dark and electrically shielded room (E:Box, Desone). Stimulus presentation was controlled from Matlab (MathWorks) using routines from the Psychophysics toolbox (Brainard, 1997). Sound levels were calibrated using a sound level meter (Model 2250, Brüel & Kjær). EEG signals were continuously recorded using an active 128-channel system (BioSemi) using Ag-AgCl electrodes mounted on an elastic cap. Four additional electrodes were placed near the outer canthi and below the eyes to obtain the electro-occulogram (EOG). Electrode offsets were kept to <25 mV. Data were acquired at a sampling rate of 1000 Hz using a low-pass filter of 208 Hz.

### EEG data analysis

Data analysis was conducted offline with MATLAB (R2017a, MathWorks), using the FieldTrip toolbox (version fieldtrip-20171001; Oostenveld et al., 2011) and custom-written routines. The data from the different blocks were preprocessed separately by bandpass filtering (0.6–70 Hz), resampling to 150 Hz, and denoising using ICA. We removed ICA components that likely reflect eye movement artifacts, localized muscle activity, or poor electrode contacts. These were identified following definitions provided in the literature (O’Beirne and Patuzzi, 1999; Hipp and Siegel, 2013). On average, we rejected 16.4 ± 1.6 components (mean ± SEM). Periods contaminated by eye blinks or movements were identified using horizontal, vertical, and radial EOG signals (Keren et al., 2010; Quax et al., 2019). We rejected trials based on a threshold of three SDs above the mean of the high pass-filtered EOGs, or during which the peak amplitude on any electrode exceeded ±175 μV. On average, we retained 93.7 ± 2.1% of trials. Also, for three participants one channel was deemed bad and interpolated with its neighbors, and for one participant two channels were interpolated. For subsequent analysis, the EEG signals were referenced to the common average reference. The analysis of eye-tracking data showed that the retained trials contained very few saccadic eye movements, suggesting that the EEG data were confounded very little by eye movements (see Results).

To extract EEG signatures of brain activity relevant for the visual motion discriminant task, we used a multivariate regularized linear discriminant analysis (LDA). We implemented an LDA to identify a projection of the EEG data in sliding windows of 92 ms duration (a window step of 46 ms) that maximally discriminated between the two directions of motion (across all three cue conditions). Within each window, the EEG activity of each electrode was averaged over time and the multivariate classifier was trained using the activity of all EEG electrodes. The data are presented such that the classification performance, or time points, noted correspond to the first time point within the classification window. Each LDA projection obtained at one time point (i.e., from one 92 ms data window) was defined by a projection vector, *w*, which describes a one-dimensional weighted combination of the EEG data (Parra et al., 2005). The regularization parameter was taken from previous work and set to 0.1. The classification performance was quantified using the area under the curve (AUC) receiver operating characteristic (ROC) based on sixfold cross-validation. Given potentially unequal trial numbers for each condition, we repeated the discriminant analysis 100 times using a random subset of 80% of the available trials for each condition, averaging the resulting AUC and projection vectors. The scalp topographies for each discriminant component (i.e., time point) were derived using the corresponding forward model defined as the normalized correlation between the discriminant component and time averaged EEG activity (Parra et al., 2005).

**Figure 2.**
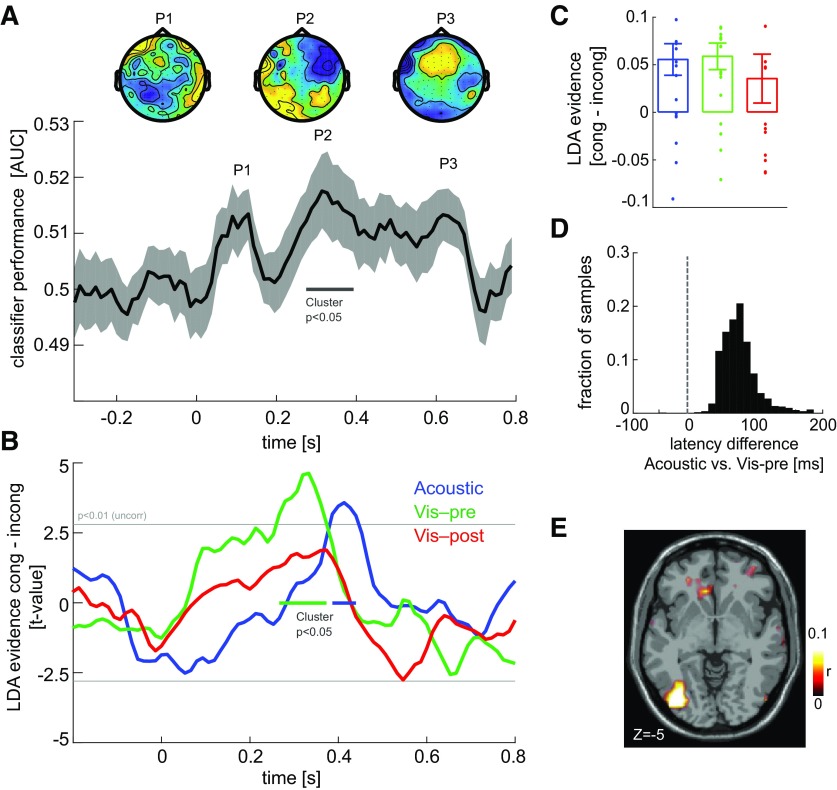
Electrophysiological correlates of cue–stimulus congruency. ***A***, Single-trial LDA was used to determine EEG components sensitive to the direction of random-dot motion, that is, sensitive to the task-relevant visual information. The curve shows the classifier performance as the area under the curve of the receiver operating characteristic (mean and SEM across participants), which was significant (cluster-based permutation test, *p* < 0.05) ∼300 ms (peak at P2). The topographies show the forward models of the LDA classifiers at three local peaks. ***B***, Effect of cue–stimulus congruency on the single-trial LDA evidence in the component derived at time P2, separately for each cue condition and shown as the group-level *t* value. Epochs with a congruency effect are indicated (cluster-based permutation test, *p* < 0.05). ***C***, Congruency effects on LDA evidence at the time points of the respective global peaks (mean and SEM across participants). ***D***, Test for a latency difference of the congruency effect in the acoustic and visual preconditions. The graph shows the group-level bootstrap distribution (2000 samples) of the latency difference between the congruency effects. ***E***, Source localization of the LDA component (at P2) determined as source-level correlation (*z*-scored) between gridwise activity and the LDA activity (group averaged; shown at MNI coordinate *z* = −5).

The discriminant output provides a measure of the single-trial evidence contained in the EEG signal about two conditions of interest. It can serve as a sensitive representation of the cerebral encoding of the task-relevant sensory information (Parra et al., 2005; Philiastides et al., 2014; Kayser et al., 2016; Grootswagers et al., 2018; Cichy et al., 2019). Following previous work, we exploited this projection to ask which specific LDA component (i.e., when in time) is affected by each of the cues. To this end, we obtained single-trial projections of the discriminant activity by applying the weights extracted at specific time points of interest (Fig. 2*A*, P1, P2, and P3) to all trials and time points. We then compared the amount of sensory evidence in the discriminant components by comparing their magnitude (ignoring the difference in sign arising from the two motion directions) between congruent and incongruent trials. This was done using sixfold cross-validation, computing LDA weights based on one subset of trials and quantifying the congruency effect on the remaining trials. Similarly, we used cross-validation when entering the LDA evidence as a predictor into a linear model of choice (or accuracy), again computing the LDA weights and the neurobehavioral regression model using distinct sets of trials.

Time–frequency representations of the prestimulus activity were obtained using wavelet analysis in FieldTrip. Frequencies ranged from 2 to 50 Hz, in steps of 1 Hz below 16 Hz and steps of 2 Hz above. The wavelet width scaled with frequency, from three cycles at 2 Hz to nine cycles above 35 Hz. To ensure that power estimates were not contaminated with stimulus-evoked responses, we zero-windowed the poststimulus period. Power estimates were *z*-scored across trials. To reduce the statistical complexity of testing for significant effects across time, frequency, and electrodes, we applied this time–frequency analysis to the one-dimensional single-trial LDA projections of interest, hence focusing on time and frequency dimensions only.

### EEG source analysis

A confirmatory source analysis was implemented by first obtaining single-trial source signals using a linear constrained minimum variance beamformer in Fieldtrip (6% normalization, using the covariance matrix obtained from −0.6 to −0.1 s prior to response). A standardized head model based on the average template brain of the Montreal Neurologic Institute (MNI) was used as single-participant MRI data were not available. Lead fields were computed using a 3D grid with 6 mm spacing. The activity at individual grid points was correlated with the linear discriminant signal over trials at the single-participant level, analogous to obtaining the forward scalp distribution via the correlation of sensor and discriminant activity (Parra et al., 2005; Haufe et al., 2014). Correlation volumes were *z*-transformed and averaged across participants.

### Statistical procedures and effect sizes

The analysis of behavioral data was based on a repeated-measures ANOVA and *post hoc* paired *t* tests. Effect sizes are reported as Cohen’s *d* for paired *t* tests and partial η^2^ (η_P_^2^) for the ANOVA (Kline, 2004). Measures of signal detection theory were derived relative to leftward motion as the “to-be-detected” stimulus. Statistical testing for perceptual weights was based on a percentile bootstrap distribution obtained by randomly resampling (2000 times) participants with replacement. Here the effect sizes are reported as Cohen’s *d* for the underlying paired *t* test. Significance testing of the single-trial discriminant performance (AUC), of congruency effects in discriminant activity, and of the influence of oscillatory power in the regression model of choice were based on group-level cluster-based permutation procedures computed by randomly permuting effect signs (Nichols and Holmes, 2002; Maris and Oostenveld, 2007). The detailed parameters were as follows: 2000 permutations; clustering bins with significant first-level tests (uncorrected at *p* < 0.05; or ROC above the 95% percentile of the distribution across bins); minimal cluster size of at least three neighbors; computing the cluster mass within each cluster; and performing a two-sided test at *p* < 0.05 on the clustered data. For these tests, we report the cluster value (*t*_sum_) of the significant cluster as effect size in addition to Cohen’s *d* derived from the univariate *t* value at the peak location.

## Results

### Cueing improves discrimination performance

Participants (*n* = 20) performed a visual motion discrimination task (leftward vs rightward random dot motion; 800 ms fixed duration) around their respective individual thresholds, as determined in an initial screening session. The different experimental conditions offered participants three types of additional cues (Fig. 1*A*). (1) During the acoustic cue condition, visual motion was accompanied by acoustic spatial motion that moved, in 66% of trials, in the same direction as the visual motion stimulus. For this task, previous work has established the electrophysiological correlates of audiovisual integration, by first extracting visual motion-sensitive EEG components using single-trial linear discriminant analysis, and then determining when this component is enhanced by congruent (vs incongruent) audiovisual information (Kayser et al., 2017; Kayser and Kayser, 2018). (2) During a visual precue condition, we provided participants with a prior visual symbolic cue that indicated the likely (66% chance of being correct) direction of the subsequent motion stimulus. This type of cue is known to facilitate perceptual performance, possibly by enhancing the encoding of the task-relevant subsequent information (Summerfield and de Lange, 2014). And last (3), during a visual postcue condition, we provided participants with a visual symbolic cue that was presented subsequent to the random dot pattern and indicated the likely (66% correct) direction of the preceding motion stimulus. While such a poststimulus cue can also facilitate performance (Bang and Rahnev, 2017), it cannot do so by influencing the encoding of visual motion information during the stimulus, and here served as a control condition for the EEG analysis.

As expected, response accuracy was significantly affected by the congruency between cue and motion stimulus. A repeated-measures ANOVA revealed no overall effect of cue (*F*_(21,19)_ = 1.17, η_P_^2^ = 0.008, *p* = 0.32; Fig. 1*B*), but a significant influence of congruency (*F*_(11,19)_ = 29.5, η_P_^22^ = 0.35, *p* < 10^−4^) and a cue–congruency interaction (*F*_(21,19)_ = 11.2, η_P_^2^ = 0.09, *p* < 10^−3^). *Post hoc* tests revealed that for each cue performance was more accurate when cue and visual motion were congruent (paired *t* tests; acoustic cue: *t*_(19)_ = 6.3, Cohen’s *d* = 1.4, *p* < 10^−5^; precue: *t*_(19)_ = 2.3, *d* = 0.5, *p* = 0.03; postcue: *t*_(19)_ = 5.1, *d* = 1.1, *p* = 0.001). Also, the congruency benefit was significantly stronger for the acoustic cue compared with both symbolic cues (*t*_(19)_ = 4.4 vs visual precue; *t*_(19)_ = 4.9 vs visual postcue; both *d* > 0.9, *p* < 10^−3^), while the two symbolic cues did not differ (*t*_(19)_ = 0.7, *d* = 0.15, *p* = 0.49).

When quantified using signal detection theory, the data revealed that sensory precision (*d*′) was significantly higher for congruent compared with incongruent trials for each cue (acoustic cue: *d*′ = 1.9 ± 0.11 vs 0.54 ± 0.14, *t*_(19)_ = 6.7, *p* < 10^−3^; precue: 1.44 ± 0.12 vs 0.92 ± 0.12, *t*_(19)_ = 2.6, *p* = 0.016; postcue: 1.60 ± 0.08 vs 1.01 ± 0.12, *t*_(19)_ = 4.56, *p* = 0.0002). To quantify the influence of the cue on response bias, we extracted the magnitude of this bias (Bang and Rahnev, 2017; acoustic cue: 0.34 ± 0.05; precue: 0.13 ± 0.05; postcue: 0.14 ± 0.03). This bias was significantly stronger for the acoustic cue compared with the symbolic cues (*t*_(19)_ = 4.6 and 4.3, Cohen’s *d* > 1.0, *p* < 10^−3^) and did not differ between the latter two (*t*_(19)_ = 0.3, *d* = 0.06, *p* = 0.75). Overall, these results show that a participant’s performance was affected by all three cue types, although the acoustic stimulus had the strongest influence both in terms of decision bias and overall accuracy benefit.

Reaction times were not affected by the cue–stimulus congruency or cue type. An ANOVA revealed no significant effects (all *p* > 0.05, maximal η_P_^2^ = 0.01); as expected, *post hoc t* tests showed only small and insignificant effects (acoustic cue: *t*_(19)_ = −1.1, Cohen’s *d* = 0.25, *p* = 0.27; precue: *t*_(19)_ = −1.5, *d* = 0.34, *p* = 0.13; postcue: *t*_(19)_ = −0.98, *d* = 0.22, *p* = 0.33).

The eye-tracking data confirmed that participants maintained the required central fixation during the stimulus period, as follows: the mean and SEM for the horizontal and vertical eye positions during the stimulus were 1.8 ± 0.5° and 1.5 ± 0.4° (*n* = 14 available participants). In addition, we found that saccadic eye movements (>0.8°) executed during the stimulus epoch were very rare (mean ± SD, 0.9 ± 1.4% of trials; maximal, 4.1%).

### Cueing does not affect temporal sampling of information

To understand whether visual motion discrimination was affected by the entire duration (800 ms) of the visual motion stimulus, or only a particular epoch of this, we computed psychophysical response templates (Neri and Heeger, 2002; Chauvin et al., 2005). This was possible as the momentary amount of evidence about the visual motion direction was manipulated randomly over time and trials, allowing us to determine the time course of the perceptual use of this information using reverse correlation (Marmarelis, 1978; Bang and Rahnev, 2017). This revealed that for all cue types motion information was significantly related to a participant’s choices for most time points throughout the stimulus, except for the first 120 ms (Fig. 1*C*, top; a group-level two-sided percentile bootstrap confidence interval across all conditions, *p* < 0.01 uncorrected). To determine whether the congruency of cue and visual motion affected the perceptual use of motion evidence, we contrasted congruent and incongruent trials for each cue separately (Fig. 1*C*, bottom). This revealed no clear difference between congruent and incongruent trials (a group-level two-sided percentile bootstrap confidence intervals was inconclusive, *p* > 0.05 uncorrected), and the overall effect size of any congruency effect was small [the peak Cohen’s *d* for the underlying paired *t* test was moderate (*d* = 0.47) compared with the overall mean effect across time and conditions (*d* = 0.18)]. This suggests that any influence of cue–stimulus congruency on response accuracy does not originate from the differential sampling of sensory information at any particular time point. This also indicates that for the analysis of the EEG data all time points are a priori equally relevant for the different conditions.

### Cueing influences the physiological correlates of visual motion encoding

To understand where and when the cues affected the sensory encoding and decision process, we first extracted signatures of visual motion encoding from the EEG data using single-trial classification. Previous work has shown that the audiovisual perceptual benefit correlates with the enhancement of the cerebral encoding of visual motion information in occipital regions ∼300 ms post-stimulus onset (Kayser et al., 2017; Kayser and Kayser, 2018). The physiological correlates of this can be extracted by first determining EEG components sensitive to visual motion direction using single-trial classification, and then determining the influence of cue–stimulus congruency on this classification component.

Here we computed a single-trial classification of visual motion direction across all cue conditions. The time course of classification performance exhibited three peaks (P1 at 0.093 s; P2 at 0.32 s; and P3 at 0.64 s; Fig. 2*A*). A statistical test based on a permutation procedure and controlling for multiple comparisons along time (at *p* < 0.05) revealed a significant cluster (P2) between 0.28 and 0.4 s (*p* = 0.014; *t*_cluster_ = 0.15). We then focused on the EEG component defined by the LDA projection obtained at P2 to ask whether the encoding of visual motion direction reflected by the underlying neural activity was significantly affected by each cue (using a cluster-based randomization procedure contrasting the LDA evidence between congruent and incongruent trials, correcting for multiple comparisons along time at *p* < 0.05; Fig. 2*B*,*C*). The visual postcue condition here served as a control, as one should not expect significant congruency effects given that the cue was presented following the stimulus. We found a significant congruency effect on visual motion encoding for the acoustic (*p* = 0.032, *t*_cluster_ =16.7, from 0.38 to 0.44 s, Cohen’s *d* at peak = 0.8) and the visual precue conditions (*p* = 0.005, *t*_cluster_ = 34.3, from 0.26 to 0.37 s, *d* = 1.0). No effect was found for the visual postcue, as expected (*p* > 0.05, *d* = 0.4). These results replicate previous findings of a sound-driven enhancement of visual motion encoding in EEG activity after 300 ms post-stimulus onset (Kayser et al., 2017; Kayser and Kayser, 2018) and demonstrate that a within-modality visual symbolic cue influences brain activity captured by the same EEG component.

The above data suggest that the congruency benefit emerges earlier for the visual precue than the acoustic cue by ∼130 ms. To corroborate this result using a direct statistical analysis, we implemented a group-level percentile bootstrap test on the difference in the latencies of the first significant congruency effects (Fig. 2*D*). The likelihood of observing a positive (i.e., visual precue being earlier) latency difference was 99.6% (i.e., *p* = 0.004 for the observed difference, with the mean of the bootstrapped distribution serving as measure of an effect size of 96 ms).

To obtain a better understanding of the cerebral sources giving rise to the motion-sensitive EEG component, we obtained a source localization of this LDA component (at time = 0.32 s). This revealed the strongest contribution from left occipital regions [Fig. 2*E*; the peak source was at MNI: −43, −78, −5 around the inferior occipital gyrus, as determined using the AAL (automated anatomical labeling) atlas], in line with the previous studies.

As a control analysis, and to determine how specific this result was for this specific EEG component (i.e., the LDA projection obtained at time = 0.32 s), we repeated the congruency analysis for the two EEG components defined by the LDAs at the two local peaks that were not significant with respect to motion direction classification (P1 and P3; Fig. 2*A*). For these, we found no significant congruency effects for any of the cue conditions (at *p* < 0.05 corrected for multiple comparisons along time for each condition, with the peak effect size across cue conditions and the two LDA components being *d* = 0.41).

### Prestimulus activity shapes stimulus–cue interactions

**Figure 3.**
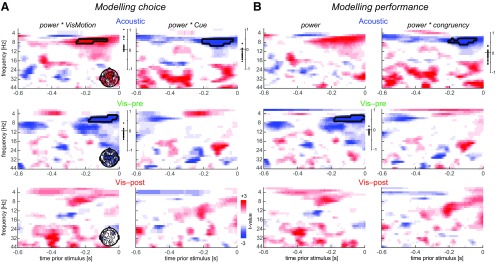
Time–frequency analysis of prestimulus influences on behavior. ***A***, For each condition, we modeled a single-trial choice based on the visual stimulus, the cue, their interaction with each other, and the interaction of each with prestimulus power. Graphs display the group-level *t* values obtained from the respective single-participant regression betas for the interaction of power with visual motion and the cue. Prestimulus activity was analyzed within the LDA component derived at P2 (Fig. 2). The topographies (insets) show the electrode-wise *t* values for the alpha band cluster in the respective panel obtained from single-electrode regression models. Cluster-averaged regression coefficients (betas) for individual participants are shown on the right. Black outlines indicate significant clusters (cluster-based permutation test, *p* < 0.05). ***B***, Same as in ***A***, but for a regression model of performance (response accuracy) based on stimulus–cue congruency, prestimulus power, and the interaction of power with congruency.

Previous work has shown that patterns of rhythmic prestimulus activity can influence how multisensory information is combined (Keil et al., 2012; Rohe et al., 2019). To understand the influence of prestimulus activity in the present paradigm, we asked whether the power of different frequency bands shapes the influence of visual motion and of the respective cue on participants’ choice. For this analysis, we derived the single-trial spectral power from the visual motion-sensitive EEG component (at P2) to avoid the statistical burden arising from a full electrode by time by frequency analysis. We then modeled participants’ single-trial responses (choice) based on a logistic GLM including the visual motion direction, the direction indicated by the respective cue, their interaction, and their interactions with prestimulus power as predictors (Fig. 3*A*). This revealed a significant contribution to participants’ behavior of visual motion, as expected per experimental design (*t*_(19)_ = 13.5, 15.9, and 16.9 for the acoustic, visual precues, and visual postcues; all at least *p* < 10^−5^, Cohen’s *d* > 3), a significant contribution of each cue confirming the above results (*t*_(19)_ = 6.6, 2.4, and 4.5 for each cue condition respectively; all *p* < 0.05, *d* = 1.4, 0.5, and 1.0, respectively), but no interaction between the visual stimulus and any of the cues (*t*_(19)_ = −0.25, −0.09, and −1.05, respectively; all *p* > 0.05, *d* ≤ 0.24).

Concerning the prestimulus activity, we found that alpha power influenced how visual motion and the acoustic cue were combined. In the auditory condition, alpha power significantly positively modulated the impact of the visual stimulus (7–8 Hz, −0.25 to −0.07 s, *p* = 0.05, *t*_cluster_ = 41, Cohen’s *d* at peak = 0.68; Fig. 3*A*) and negatively modulated the influence of the acoustic cue (7–9 Hz, −0.2 to −0.03 s, *p* = 0.023, *t*_cluster_ = −65, *d* = 0.88). Across individuals, the positive and negative alpha effects (i.e., the regression betas) were anticorrelated (*r*_(19)_ = −0.45, *p* = 0.038, Spearman rank correlation), suggesting that these arise from a common neural generator. This result was confirmed in a separate model, where we predicted performance (response accuracy, rather than choice) based on prestimulus power and its interaction with the congruency of visual motion and the acoustic cue (Fig. 3*B*). This revealed no effect of power (no significant cluster, maximal *d* = 0.53) but a significant interaction of power and congruency in the alpha band, as expected (*p* = 0.045, *t*_cluster_ = −50, *d* = 0.8).

For the visual precue alpha activity modulated how visual motion shaped behavior (5–7 Hz, −0.18 to −0.01 s, *p* = 0.029, *t*_cluster_ = −53, *d* = 0.82), but there was no significant interaction of prestimulus activity with the cue (maximal *d* = 0.23). The influence of alpha band activity on behavior in this condition was hence restricted to an influence of alpha power on how the visual information shaped responses, but did not depend on the congruency between visual motion and the cue. The latter conclusion was also confirmed by an absence of a modulatory influence of alpha power on congruency in a direct regression model of performance on power and congruency. Here we confirmed the expected influence of alpha power (Fig. 3*B*; *p* = 0.022, *t*_cluster_ = −64, *d* = 0.79) and found no effect of the interaction (no significant cluster, maximal *d* = 0.61). Because the cue preceeded the time window in which prestimulus activity was quantified, we also asked whether the symbolic cue had a direct influence on oscillatory power: we contrasted alpha power between the two directions of the visual precue but found no significant effect (*t*_(19)_ = 0.64, *p* > 0.05, *d* = 0.004), suggesting that the visual precue did not influence the subsequent alpha band activity. Finally, and as expected, for the visual postcue condition there were no significant effects (all *p* > 0.05, maximal *d* = 0.55).

Given that the analyzed LDA component reflects a mixture of different EEG sensors, we also computed these regression models at the sensor level, focusing only on the alpha frequencies and time bins revealed by the above analysis (Fig. 3*A*, insets). This confirmed a contribution of left occipital and frontocentral electrodes to the alpha effect.

To better understand how alpha activity influences behavioral responses in the audiovisual condition, we sorted trials into groups composed of particularly low and high alpha power (median split; using the intersection of the two alpha clusters for the acoustic cue condition from Fig. 3*A*) and compared how alpha related to response accuracy depending on stimulus–cue congruency. That is, we tested how behavioral performance covaries with alpha power in congruent and incongruent trials. For the acoustic cue, this revealed a significant dependence of performance on alpha power in incongruent trials (*t*_(19)_ = 2.8, *d* = 0.62, adjusted *p* = 0.04, paired two-sided *t* test, false discovery rate adjusted for multiple comparisons using the Benjamini–Hochberg procedure), but not in congruent trials (*t*_(19)_ = 0.69, *d* = 0.15, adjusted *p* = 0.39). This suggests that the influence of the acoustic cue is shaped by alpha power particularly when the cue mismatches the visual information. For the visual precue, there was no significant dependence of performance on alpha power in either trial type (*t*_(19)_ < 1.8, adjusted *p* > 0.05), in line with the above null finding for an interaction of alpha power and the symbolic cue.

## Discussion

Both abstract sensory cues inducing specific expectations as well as multisensory information can improve perceptual performance. Using a visual motion discrimination task we here compared the effect of within-modality symbolic cues and multisensory combination. The behavioral data showed that both a prior visual symbolic cue and a visual symbolic cue following the task-relevant stimulus can induce similar behavioral benefits, which is in line with previous work (Bang and Rahnev, 2017). However, the congruency benefit from a congruent acoustic cue was significantly stronger compared with these symbolic cues, although all cue types had the same level of accuracy in predicting the correct visual motion direction, and the visual precue was already presented before the task stimulus. It remains to be investigated whether there is a genuine benefit for multisensory information, or whether potential differences in attention for within-modality and between-modality associations contributed to these differences in cue efficiency (Talsma et al., 2010).

### Multisensory and expectation-based facilitation of behavior

We asked whether the perceptual benefit arising from a prior symbolic cue and from multisensory information arise from similar neurophysiological correlates. Concerning the acoustic cue, the present data reproduce previous findings, in that congruent acoustic information facilitates the encoding of visual motion in occipital brain regions at latencies of ∼300 ms post-stimulus onset. These results support the notion of multisensory influences in low-level sensory regions (Rohe and Noppeney, 2015; Cao et al., 2019), fit with previous neuroimaging studies reporting multisensory influences in visual motion regions (Poirier et al., 2005; Lewis and Noppeney, 2010; Alink et al., 2012), and directly reproduce previous results obtained using a very similar task (Kayser et al., 2017; Kayser and Kayser, 2018). The minor differences in the stimulus classification performance obtained here and in that previous study may have two potential explanations: first, we here used a stimulus with dynamic motion coherence, as opposed to the fixed motion coherence used previously (Kayser et al., 2017; Kayser and Kayser, 2018). Second, the present task emphasized response accuracy while the previous study used speeded reactions.

Concerning the symbolic cue, our results suggest that the benefits induced by priming and multisensory information at least partly arise from a shared neural substrate. This conclusion is supported by the observation that the same visual motion-sensitive EEG component was affected by a prior visual cue and by multisensory congruency. Still, the priming benefit emerged significantly earlier in time. Possibly, the information provided by the symbolic cue was already fully processed at the time of visual motion onset, while the acoustic cue was presented simultaneously with the visual motion. Previous studies have shown that expectation can influence the activity of neurons in regions processing visual motion (Schlack and Albright, 2007; Kok et al., 2013), which is in line with the localization of the cue–stimulus congruency benefit in the present study to an occipital EEG component that is sensitive to visual motion.

We did not include a neutral condition, that is, a sound not inducing any motion percept or a neutral symbolic cue. As a result, the present study cannot dissociate whether congruent or incongruent cues enhance or reduce performance compared with a baseline condition (de Lange et al., 2013; Bang and Rahnev, 2017). While a neutral condition is required to judge the specific influence of each individual cue, and to understand how predictive versus unpredictive cues influence cerebral processing, the comparison of between-modality and within-modality cues, which is of relevance here, is not affected by the lack of a neutral condition.

Expectation has been suggested to act via two types of mechanisms, one reflecting a change in postsensory decision criteria and one reflecting an improvement in the encoding of sensory information in low-level brain regions (Summerfield and Egner, 2009; Summerfield and de Lange, 2014; Bang and Rahnev, 2017; Rungratsameetaweemana et al., 2018). The behavioral results obtained from the visual postcue condition confirm the relevance of postsensory contributions: in this condition, the cue was presented only after the offset of the task-relevant stimulus, but the perceptual benefit was comparable to the prior visual symbolic cue. At the same time, our data provide a direct cerebral correlate of the benefit induced by the prior symbolic cue in a generator of physiological activity that can be interpreted as arising from low-level (visual motion-encoding) brain regions. The latency of the congruency effect was >200 ms, making it difficult to speak of early (short-latency) neural effects, and leaving the possibility that this congruency benefit arises in a top–down manner.

Importantly, the same question of whether a benefit induced by auxiliary information arises from sensory- or decision-level mechanisms is discussed in the context of multisensory perception (Bizley et al., 2016). Recent work has shown that multisensory integration arises in a hierarchical and distributed manner, which makes it difficult to pinpoint a specific single underlying mechanisms or brain region underlying this (Rohe and Noppeney, 2015; Aller and Noppeney, 2019; Cao et al., 2019; Rohe et al., 2019). A similar conclusion may hold for general cueing effects. Hence, a parsimonious interpretation is that both sensory-level and postsensory mechanisms contribute to the symbolic cueing benefit in the present paradigm, with the sensory-level contribution arising from neural processes that are also susceptible to multisensory influences.

### Auxiliary multisensory effects and cueing

In the literature on multisensory perception, two types of multisensory effects have been described. On the one hand, there is sensory integration, also known as fusion, where the partly redundant task-informative evidence from two modalities is combined, possibly in a bottom–up manner (Ernst and Bülthoff, 2004; Angelaki et al., 2009; Cao et al., 2019). On the other hand, so-called auxiliary multisensory interactions have been described. Here, the perception of a task-relevant stimulus is affected by the presentation of a stimulus in another modality, which by itself does not offer primary task-relevant information (Odgaard et al., 2003; Jaekl and Harris, 2009; Caclin et al., 2011; Chen and Spence, 2011; Gleiss and Kayser, 2014a,b). One example is the enhanced contrast sensitivity when a visual stimulus is accompanied by an irrelevant sound (Lippert et al., 2007). In these paradigms, the auxiliary stimulus could be interpreted as an additional “cue” that does not carry information about the specific task-relevant variable (e.g., motion direction or stimulus lateralization) but carries other information, such as about stimulus timing, that nevertheless helps to facilitate performance.

Interestingly, one study (Kim et al., 2012) reported such an auxiliary multisensory interaction in the context of visual motion detection: this study showed that the detection of which of two stimulus intervals carries coherent (as opposed to random) motion can be enhanced when both intervals are accompanied by the same acoustic motion. Although the sound was identical across stimulus intervals, performance was significantly enhanced when the sound was congruent to the visual motion. One could interpret this benefit as resulting from the enhancement of visual motion encoding by acoustic information in a bottom-up manner (Kim et al., 2012). However, an EEG study capitalizing on the same paradigm linked the behavioral improvement to changes in poststimulus parietal alpha-band activity, which is most parsimoniously understood as reflecting an attentional effect (Gleiss and Kayser, 2014b). In line with this, several auxiliary multisensory influences have been considered to arise from changes in sensory saliency or attentional gain (Lippert et al., 2007; Chen and Spence, 2011; Gleiss and Kayser, 2014a).

The present paradigm differed from this two-interval task (Kim et al., 2012) in that the additional multisensory or symbolic cues were directly predictive of the likely motion direction, and hence these could be used in principle (by guessing in proportion to the cue validity) to solve the task. In that sense, the present paradigm more directly corresponds to a sensory integration task, where one task-relevant stimulus (here visual motion) is accompanied by a second task-informative stimulus (here the cue). Further work is required to better understand which perceptual and neural mechanisms are common to abstract cueing, multisensory fusion, and other types of multisensory effects, and which perceptual and neural mechanisms differentiate these paradigms. The analysis used here could provide one experimental approach to address this question in the future.

### Prestimulus brain state and cue combination

To understand how prestimulus activity influences the manner in which two pieces of sensory evidence are combined, we modeled the influence of oscillatory power on how visual motion and the cue are combined. This revealed that alpha power significantly interacted with the impact of visual motion on behavior regardless of the nature of the cue, and was predictive of how an acoustic but not a visual symbolic cue influenced behavior. In the multisensory condition, stronger alpha power apparently enhances the influence of the visual stimulus but reduces the influence of the sound stimulus, seemingly reflecting a relative weighting of the two stimuli. This apparent influence of prestimulus activity was stronger for incongruent trials, where a participant’s accuracy was worse when alpha power was low. This is in line with the recent suggestion that reduced prestimulus parietal alpha reflects the a priori tendency with which participants combine multisensory information (Rohe et al., 2019). With this interpretation in mind, the analyzed alpha band activity is reflective of a high-level amodal process arbitrating between sensory integration and segregation.

In the visual precue condition, reduced alpha power interacted with the influence of the visual stimulus, in line with reduced alpha power reflecting enhanced visual attention (Thut et al., 2006; Romei et al., 2007). However, we did not find a significant interaction of alpha power with the visual symbolic cue, nor was alpha power itself directly influenced by the preceding cue. This is in contrast to previous studies proposing a role of prestimulus alpha power in mediating the influence of prior expectations (Bauer et al., 2014; Mayer et al., 2016; Samaha et al., 2018). One possibility is that alpha power mediates expectations most strongly for spatial or temporal stimuli (Worden et al., 2000; Samaha et al., 2015, 2016), although effects for symbolic cues have been reported as well (Mayer et al., 2016). Another possibility is that the analyzed alpha activity in the two conditions originated from distinct neural structures. This may seem unlikely given that the analysis of spectral activity was based on the same electrode combination (“virtual signal”) for both conditions, and given that the respective sensor-level topographies were similar between the two cue types. However, it remains debated whether parietal alpha band activity arises from a single supramodal mechanism or distinct and modality-specific generators (Banerjee et al., 2011). Previous work has linked parietal alpha power to multiple sensory or cognitive processes, including spatial attention or sensory arbitration (Thut et al., 2006; Romei et al., 2007; Rohe et al., 2019), without clear evidence for how to experimentally separate these potentially different mechanisms reflected in the same physiological activity pattern. As a result, future work specifically needs to address the relevance of attention and alpha band activity when investigating the possibly shared or distinct mechanisms underlying abstract cueing, multisensory fusion, and other types of multisensory effects (Summerfield and Egner, 2009; Talsma et al., 2010; Zuanazzi and Noppeney, 2019).

## Synthesis

Reviewing Editor: Leonard Maler, University of Ottawa Decisions are customarily a result of the Reviewing Editor and the peer reviewers coming together and discussing their recommendations until a consensus is reached. When revisions are invited, a fact-based synthesis statement explaining their decision and outlining what is needed to prepare a revision will be listed below. The following reviewer(s) agreed to reveal their identity: Patrick Bruns.

Editorial comments

Behaviour and perception can be improved by combining different sense inputs and by prior knowledge related to the task. The question addressed in this interesting Ms is whether the improvements are due to similar or distinct mechanisms. The subject is important and the research presented interesting, but both reviewers noted that the data and analyses may not be adequate for clean and conclusive conclusion. Below, I include an edited version of the reviewer comments. These were very clearly presented and relate to the statistical design and analyses.

Please address all these points in a revision.

Reviewer #1

The paper compared multisensory vs. expectation-based facilitation of visual motion direction discrimination in an EEG experiment. Three conditions were tested, one in which visual motion stimuli were accompanied by auditory motion stimuli (66% congruent), one in which a symbolic visual cue indicated the likely motion direction prior to the motion stimulus, and one in which the symbolic cue was presented after the motion stimulus. All three cues enhanced accuracy in congruent vs. incongruent trials, but to a larger extent with the auditory cue than with the symbolic visual cues. The EEG analysis revealed a component that was sensitive to visual motion direction (P2) and was also modulated by congruency of the auditory as well as the pre-stimulus symbolic cues. The authors interpret this finding as evidence for a shared underlying process of multisensory integration and within-modality cuing effects on visual motion discrimination. In addition, it was found that pre-stimulus alpha activity was related to weighting of the auditory cue but not to the behavioral influence of the symbolic cue.

a) It is not clear whether the auditory condition really measures effects of multisensory integration. It could be that participants simply extracted the motion direction from the auditory stimulus and used it as an abstract cue for the likely visual motion direction without actually integrating visual and auditory motion information at a sensory level. In this case, the result of a shared mechanism would be somewhat trivial. There is some evidence from better controlled studies (e.g., Kim et al., Psychol Sci, 2012) suggesting that auditory and visual motion cues similar to the ones used in the present study are integrated, but how can we be sure that this was indeed the case in the present study? The observed EEG effects are rather late and thus do not exclude a decisional rather than perceptual influence on visual motion perception.

b) There was no control condition without cuing. Thus, it is unclear whether (relative to baseline) performance increased with a congruent cue, decreased with an incongruent cue, or both.

“For example, the overall tendency to combine visual and acoustic cues in a ventriloquist paradigm is influenced by the level of pre‐stimulus alpha power (Rohe et al. 2019).” (p. 2 bottom). Note that Rohe et al. (2019) did not use the ventriloquist paradigm, in this paper they investigated the sound-induced flash illusion.

Method section indicates that “data were obtained from a planned sample of 20 healthy adult participants” (p. 3 top), but it is not clear on which a priori criterion (power analysis, for which statistical test and which effect size?) the sample size of 20 was based.

Statistical procedure subsection (p. 6) says that “effect sizes are reported as Cohen’s d’ for paired t‐tests and eta‐squared for the ANOVA”. Note that it is Cohen’s d (just the letter without the prime), please correct throughout the manuscript. Repeated-measures ANOVA effect sizes should be reported as either partial eta squared or as generalized eta squared. It is not clear which version of eta squared was used here, please clarify.

ANOVA F-values should be reported with both numerator and denominator degrees of freedom, but manuscript reports F-values only with numerator dfs in parantheses, please correct. Also all t values should be reported with their dfs, this is not always done.

“In different blocks we offered participants three types of additional cues” (p. 7 top). Earlier it is claimed that trials were “grouped into four experimental blocks, each comprising all three conditions” (p. 3 bottom). Which is correct, block-wise manipulation or intermixed presentation of cue conditions?

Coherence changed for “every period of 12 consecutive frames”: was this a sliding window or after 12, 24, 36 frames a.s.f.?

Effect of pre-stimulus alpha on cue effect: the auditory cue followed the pre-stimulus period, but the visual cue occurred at the same time at which pre-stimulus alpha is measured. Couldn’t the visual cue already have influenced the pre-stimulus alpha, which might explain why there was no (additional) interaction of pre-stimulus activity with cue in this condition?

Reviewer #2

Some of the results of this study replicate previous finings, however there are important novel observations which can potentially advance the field.:

a) Page 4. I was delighted to see, that the authors carefully controlled for eye movements. It is indeed crucial in experimental paradigms where some spatial aspect of the stimulus is manipulated, and this information is decoded from the neural activity (see. Quax et al. 2019, Neuroimage). I was a little bit surprised however, that the authors did not use the generally superior information at hand from the eyetracking when rejecting recording periods based on eyeblinks or saccades and used the EOG instead. Could the authors please justify their decision?

b) Page 5. The authors describe that the LDA analysis was performed in windows of 92 ms, however there are some important details missing here. Based the smoothness of the plot in Figure 2A I assume that this was a sliding window analysis, i.e. the window was shifted across the whole trial from time point to time point? Within the 92 ms window where was the onset of the window registered? This is very important in the interpretation of the onset times of effects as with windowed analyses information from later time points can bleed forward in time depending where the onset is registered within the window (see. Grootswagers et al. 2017, JoCN). It is also unclear how the data are treated within the window: are the topographies averaged or concatenated? Given that later the weights are converted to the corresponding forward model activations I assume the topographies were averaged within the window, so the weights remained interpretable this way. Nevertheless, all these details should be part of the methods section.

c) Page 9. I was a little bit surprised that only the second peak of the decoding accuracy was significant. Looking up the referenced previous results in (Kayser et al. 2017; Kayser & Kayser 2018) there seem to be an earlier and/or a later significant peak present. I was wondering if this is simply a difference in signal to noise between the studies (i.e. maybe smaller number of trials) or could it be related to the introduction of the within-modality cues?

d) Page 11. “We then modelled participants single trial responses...” I had a bit of difficulty understanding weather ‘the cue’ in this sentence refers to the cue type or the cued motion direction. I think it would be beneficial to change ‘cue’ to ‘cued direction’ or something similar to make it unambiguous. Also please update Figure 3 correspondingly.

e) Page 11. Regarding the pre-stimulus spectral results, I am a little bit confused by the opposing effects of alpha power on visual motion between the acoustic and Vis-pre cues. As the authors point out in the discussion there is evidence, that reduced alpha reflects enhanced visual attention, which would predict exactly the opposite of the observed effects in case of an acoustic cue: low alpha power would be associated with enhanced influence of the visual stimulus and high alpha power with enhanced influence of the (acoustic) cue. This is clearly not the case and I wonder if the authors could reflect on this?

f) Page 13. “...although all cue types had the same level of reliability,...” I believe the authors refer to the probability with which the cue represents the correct motion direction under ‘reliability’. If yes, then the correct word to use here would be ‘accuracy’ as ‘reliability’ is usually referred to as the inverse variance of the probability distribution of the sensory signal in the multisensory literature, hence it is confusing to use in the above context.

g) Page 14. Discussion of pre-stimulus results: I felt that the usage of the words ‘influenced’, ‘boosted’, ‘enhanced’, ‘reduced’ imply a causal role of alpha oscillations in the results, which I believe cannot be concluded from the analysis at hand. Could the authors please use words which emphasise the correlative nature of these results.

h) Minor typos:

Page 3: LCD monitor instead of LDC

There is an extra ‘using’ in the sentence describing the staircase procedure

Page 14: “...stronger alpha power boosted in the influence...”, the ‘in’ is unnecessary
